# Progressive Upsampling Generative Adversarial Network with Collaborative Attention for Single-Image Super-Resolution

**DOI:** 10.3390/jimaging12020079

**Published:** 2026-02-11

**Authors:** Haoxiang Lu, Jing Zhang, Mengyuan Jing, Ziming Wang, Wenhao Wang

**Affiliations:** 1 Guangdong Cardiovascular Institute, Guangdong Provincial People’s Hospital, Guangdong Academy of Sciences, Guangzhou 510080, China; hxlu1005@163.com (H.L.); jingmengyuan@gdph.org.cn (M.J.); 2 Department of Radiology, Guangdong Provincial People’s Hospital, Guangdong Academy of Medical Sciences, Southern Medical University, Guangzhou 510080, China; 3 Guangdong Provincial Key Laboratory of Artificial Intelligence in Medical Image Analysis and Application, Guangzhou 510080, China; 4 School of Computer and Information Security, Guilin University of Electronic Technology, Guilin 541004, China; worthyman@guet.edu.cn (Z.W.); whwang2018@163.com (W.W.); 5 School of Business, Guilin Institute of Information Technology, Guilin 541100, China

**Keywords:** single-image super-resolution, generative adversarial network, progressive upsampling, attention mechanism

## Abstract

Single-image super-resolution (SISR) is an essential low-level visual task that aims to produce high-resolution images from low-resolution inputs. However, most existing SISR methods heavily rely on ideal degradation kernels and rarely consider the actual noise distribution. To tackle these issues, this paper presents a progressive upsampling generative adversarial network with collaborative attention mechanism called PUGAN. Specifically, the residual multiscale blocks (RMBs) based on stacked mixed-pooling multiscale structures (MPMSs) is designed to make full use of multiscale global–local hierarchical features, and the frequency collaborative attention mechanism (CAM) is used to fully dig up high- and low-frequency characteristics. Meanwhile, we design a progressive upsampling strategy to guide the model’s learning better while reducing the model’s complexity. Finally, the discriminator is also used to evaluate the reconstructed high-resolution images for balancing super-resolution reconstruction and detail enhancement. Our PUGAN can yield comparable PSNR/SSIM/LPIPS values for the NTIRE 2020, Urban 100, and B100 datasets, whose values are 33.987/0.9673/0.1210, 32.966/0.9483/0.1431, and 33.627/0.9546/0.1354 for the scale factor of ×2 as well as 26.349/0.8721/0.1975, 26.110/0.8614/0.1983, and 26.306/0.8803/0.1978 for the scale factor of ×4, respectively. Extensive experiments demonstrate that our PUGAN outperforms state-of-the-art SISR methods in qualitative and quantitative assessments for the SISR task. Additionally, our PUGAN shows the potential benefits to pathological image super-resolution.

## 1. Introduction

Low-resolution (LR) images encounter uncomfortable visual quality (e.g., blurry details and outlines, low peak signal-to-noise ratio, etc.) and compromised unreliable delivery of information [1,2]. The former brings about an unsatisfactory visual experience, while the latter may be harmful to the advanced visual tasks, such as inaccurate image understanding and object detection. Therefore, promoting the resolution of the images has proven highly effective for image understanding. Single-image super-resolution (SISR) can reconstruct a visually high-resolution (HR) images with clearer details and outlines from the corresponding LR versions, which has been widely applied in computer-aided diagnosis (CAD) [3], advanced driver assistance systems (ADAS) [4], remote sensing [5,6], and other real-world practical applications. Pathological images contain tumor microenvironment (TME), including tumor epithelial, tumor-infiltrating lymphocytes (TILs), tumor-associated stroma, etc., clinically related to the occurrence, development, and metastasis of tumors. In clinical diagnosis, pathologists conduct a step-by-step analysis of the pathological images from low magnification to high magnification. But the scanning of high-magnification pathological images is time-consuming and labor-intensive, and the storage is also challenging. Many researchers [7,8,9] have proven that the SISR technology can yield high-magnification pathological images from their low-magnification versions, whereas the inherently ill-posed nature of SISR makes it a challenging problem.

In earlier times, interpolation-based [10], reconstruction-based [11,12], example-based [11], dictionary learning-based [13,14], and other traditional SSIR approaches have been proposed for yielding HR images. Among them, the interpolation-based methods [10] (such as bilinear interpolation, bicubic interpolation, etc.) and the reconstruction-based methods (such as maximum a posteriori probability estimation, convex set projection, etc.) can estimate the pixels of the HR images based on the LR inputs, but they encounter poor robustness. The latter two methods struggle to construct the knowledge base regarding the mapping from the LR inputs to their HR versions. However, they typically contain resource-intensive operations and inevitably introduce observable handcraft-halos in the HR images. Benefiting from the rapid development of deep learning technology and computing resources, many researchers have proposed learning-based SISR approaches, including convolutional neural networks (CNNs) [15,16], generative adversarial networks (GANs) [17,18], diffusion model [19], etc., for exploring a reliable nonlinear mapping between the LR images and their corresponding HR ones. For example, Dong et al. [15] first illustrated that traditional sparse-coding-based SR methods can be regarded as a deep CNN, and further presented a CNN-based SISR method to directly learn an end-to-end mapping between the LR/HR paired images. Umer et al. [20] employed an adversarial strategy to train the model with pixel-wise supervision in the HR domain from its generated LR counterpart. Shang et al. [16] further proposed a diffusion probabilistic model (DPM) based on residual structure, which utilizes a CNN to restore primary low-frequency components and a DPM to predict the residual between the ground truth image and the CNN-predicted image. Although most of learning-based SISR approaches can generate visually pleasing HR images, they inevitably generate artifact-halos and blurry details due to the ideal degradation means (i.e., bicubic down-sampled, etc.) and rarely take the real noise (i.e., inherent sensor noise, stochastic noise, etc.) into account.

To solve the above-listed problems, we present a feasible and effective SISR method named PUGAN. This SISR method contains three main components: a noise collection and frequency decomposition, a frequency collaborative progressive generator (FCPG), and an image perceptual discriminator (IPD). In the first stage, a sliding window is employed to collect noise from the LR input to construct a noise pool, and the convolutional Gaussian filtering is used to extract the high- and low-frequency information of the input. In the FCGN stage, we introduce some residual multiscale blocks (RMBs) based on the mixed-pooling multiscale structure (MPMS) to fully explore multiscale features at global or local levels, and the collaborative attention mechanism (CAM) can make full use of the complementarity of high- and low-frequency information. Meanwhile, we introduce a progressive upsampling strategy to better guide the model’s learning while reducing the model’s complexity. In the IPG stage, the discriminator is hired to evaluate the reconstructed HR images for balancing super-resolution reconstruction and detail enhancement. The validation experiments conducted on the public benchmarks have demonstrated that our proposed method works better than state-of-the-art SISR methods.

The main contributions of this work are summarized as follows:

(1) We propose a progressive upsampling generative adversarial network with collaborative attention (called PUGAN) for the SISR task. Extensive experiments demonstrate that our method achieves the state-of-the-art performance and enjoys high efficiency. Meanwhile, our PUGAN also generalizes well to pathological images.

(2) We design an FCPG containing RMBs and CAM to generate the HR images. The RMB built upon MPMS in a dual-patch manner to fully explore multiscale global and local features, further enhancing the model’s multiscale representation capability. Meanwhile, the CAM is employed to explore the complementarity of high- and low-frequency features.

(3) We construct a noise pool using the sliding window to collect noise from the LR input and further select noise randomly to simulate real noise. Meanwhile, the progressive upsampling strategy is further introduced to our PUGAN to guide the model’s learning better while reducing the model’s complexity.

We demonstrate the organization of the remainder in this paper as follows. In Section 2, we review the previous work related to our method, including learning-based SISR methods and attention mechanisms. In Section 3, the motivation and architecture of our proposed method are presented in detail. In Section 4, we give the implementation details and experimental settings, as well as an ablation study and comparisons with state-of-the-art SISR methods. In the end, we present the conclusion of our work in Section 5.

## 2. Related Works

Over the past decade, many SISR approaches have been proposed. We outline some related learning-based SISR methods and attention mechanisms in this section.

### 2.1. Learning-Based SISR Methods

Ranging from the first SISR network SRCNN [15], learning-based SISR approaches relying on the powerful feature extraction and representation capability have spurred dramatic improvements from different perspectives. Lim et al. [21] injected the residual learning techniques into the CNN to develop an enhanced deep SR network (EDSR) for reconstructing HR images of different upscaling factors in a single model. Kong et al. [22] further proposed a ClassSR to improve image resolution using different methods based on the difficulty of sub-images. However, these CNN-based methods primarily focus on local structures and details, inevitably leading to unclear details in some enhanced images. Hence, Zhao et al. [23] employed a spatial shuffle multi-head self-attention for global pixel dependency modeling. Li et al. [24] employed the high-frequency enhancement residual block to extract high-frequency information, and further enhanced the global and local features by the shift rectangle window attention and the hybrid fusion blocks. These have proven that the vision transformer (ViT) can capture global information by effectively extracting long-range dependencies, while overlooking the importance of high-frequency features. So, Wu et al. [25] combined CNN with transformer to propose a hybrid SP-MISR network for sequential images with fixed sub-pixel shifts. Liu et al. [26] proposed a hybrid Mamba–Transformer model for SISR for effectively leveraging both Mamba and Transformer architectures. However, these methods confront a heavy computational burden and overly rely on paired images, limiting their real-world applications.

To reduce the reliance on synthetic datasets, Wang et al. [27] proposed an enhanced super-resolution GAN (ESRGAN) based on the residual-in-residual dense block (RRDB) without batch normalization, adversarial loss, and perceptual loss. Notably, the LR images obtained through known degradation (for instance, bicubic downsampling) from the HR images cannot accurately describe the real image degradation. To tackle this issue, Prajapati et al. [28] designed a new direct unsupervised super-resolution method using GAN (called DUS-GAN) to accomplish the SR task without degradation estimation of real-world LR data. Ma et al. [18] presented a detail-enhanced generative adversarial network to better reconstruct the image details even with a limited number of training samples. Dong et al. [29] performed a cross dropout-based dynamic network (CDDNet), using the degradation of LR images using degradation weights as the global attention, for multi-degradation blind SR. Cho et al. [30] leveraged the connection between degradation kernel shapes and the frequency-domain characteristics of LR images to simplify the kernel estimation process.

### 2.2. Attention Mechanism

In human perception, attention is the process by which the visual system prioritizes a sequence of information, selectively focusing on salient stimuli [31,32]. Inspired by this mechanism, some researchers proposed attention mechanisms to further promote the feature extraction and representation ability of learning-based methods, which have been widely applied in natural language processing, medical image analysis, natural image processing, and other computer vision applications [33,34,35]. Park et al. [36] proposed the bottlenet attention module (BAM), which dynamically selects features in a self-inclusion and adaptive manner. Subsequently, the convolutional block attention module (CBAM) [37] was proposed by combining channel attention with spatial attention to explore the relationship between internal channels and enhance the implicit information correlation. Zhang et al. [38] integrated the attention mechanism with the residual idea to design the residual channel attention mechanism module to optimize the model’s ability to extract details. Sun et al. [35] replaced the ordinary convolutions in the attention mechanism module with multiscale dilated convolution to make it extract feature images at different receptive field scales. Wang et al. [39] designed a multiscale large kernel attention (MLKA), including multiscale structure and gate schemes to obtain the abundant attention map at various granularity levels. Wu et al. [40] integrated the channel attention and self-attention mechanisms to design a hybrid attention calibration mechanism. Guo et al. [41] developed a hybrid attention-dense connected Transformer network containing an effective dense Transformer block (EDTB) and a hybrid attention block (HAB) for effective performance on SR tasks with magnification factors of 2, 3, and 4. Malkocoglu et al. [42] designed a deep channel attention SR model for promoting the performance of object detection. Su et al. [43] introduced a concise yet effective soft thresholding operation to obtain high-similarity-pass attention. Zhang et al. [44] proposed an efficient shuffle attention (SA) module to address this issue, which adopts Shuffle Units to combine two types of attention mechanisms effectively. However, most of the existing attention modules suffer from low computational efficiency and rarely consider valuable structural priors.

## 3. Methodology

In this section, we first briefly introduce the overall network architecture of our proposed PUGAN. Subsequently, the noise collection and frequency decomposition, FCPG, IPD, as well as loss functions are demonstrated in detail.

### 3.1. Network Architecture

**Figure 1:**
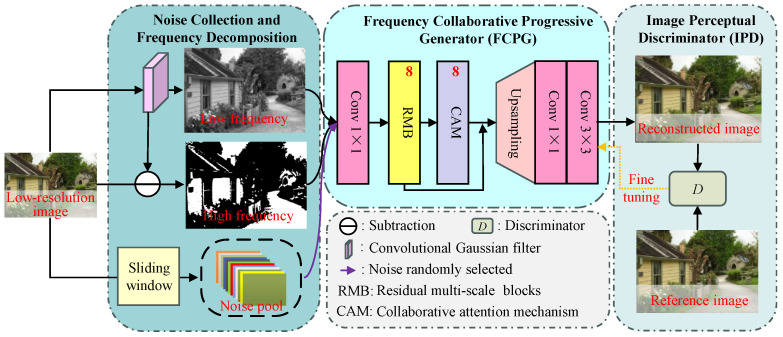
The workflow of our PUGAN. In first stage, the sliding window is used to construct noise pool and further extract high/low-frequency information from the LR input. In our FCPG, it first employs eight residual multiscale blocks (RMBs) built upon a mixed-pooling multiscale structure (MPMS) to fully explore multiscale local–global features. Then, eight collaborative attention mechanism (CAM) blocks are further used to utilize the complementarity of high/low-frequency information. In our IPG, we employ a discriminator to balance super-resolution reconstruction and detail enhancement.

This paper proposes a progressive upsampling generative adversarial network with collaborative attention named PUGAN, and the workflow of our PUGAN is demonstrated in Figure 1. Our PUGAN involves three main components: noise collection and frequency decomposition, FCPG, and IPD. Following reference [45], we introduce a discriminator network $D_{\theta_{D}}$, which is optimized alternately with the generator $G_{\theta_{G}}$ to solve the adversarial min–max problem:

(1) $$\min_{\theta_{G}}\max_{\theta_{D}}E_{I^{\mathrm{HR}}∼p_{\mathrm{train}}\left(I^{\mathrm{HR}}\right)}\left[\log D_{\theta_{D}}\left(I^{\mathrm{HR}}\right)\right]+E_{I^{\mathrm{LR}}∼p_{G}\left(I^{\mathrm{LR}}\right)}\left[\log\left(1-D_{\theta_{D}}\left(G_{\theta_{G}}\left(I^{\mathrm{LR}}\right)\right)\right)\right]$$

The core idea of this formulation is to train a generative model *G* to deceive a differentiable discriminator *D*, which is itself trained to distinguish super-resolved images from real ones. Therefore, *G* learns to produce outputs so realistic that they become difficult for *D* to classify.

### 3.2. Noise Collection and Frequency Decomposition

Currently, most existing SISR methods usually adopt the ideal bicubic sampling as the degradation kernels, which cannot accurately describe the image degradation, resulting in artifact-halos in some reconstructed HR images. Hence, inspired by [46,47], we perform a slide window to collect noise from the LR input, and we further randomly select noise to simulate the distribution of the image noise captured in the real world. Specifically, given a global patch $P_{i}$ with the size of d×d and a local patch $Q_{j}^{i}$ (notably, each global patch $P_{i}$ is extracted by scanning the noisy LR input with a stride $s_{g}$, and each $Q_{j}^{i}$ can be obtained by scanning inside $P_{i}$ with a stride $s_{l}$). We determine whether a given $P_{i}$ is a smooth patch by evaluating the differences in mean and variance between $P_{i}$ and each of its local patches $Q_{j}^{i}$. More precisely, two constraints are first defined as follows:

(2) $$\left\{\begin{array}{c} \left|Mean\left(Q_{j}^{i}\right)-Mean\left(P_{i}\right)\right|\leq \alpha \cdot Mean\left(P_{i}\right)\\ \left|Var\left(Q_{j}^{i}\right)-Var\left(P_{i}\right)\right|\leq \beta \cdot Var\left(P_{i}\right) \end{array}\right.$$

where $Mean\left(\cdot \right)$ and $Var\left(\cdot \right)$ calculate the mean and variance of a global patch $P_{i}$ and a local patch $Q_{j}^{i}$, and $\alpha ,\beta \in \left(0,1\right)$. If both constraints are simultaneously satisfied for every local patch $Q_{j}^{i}$, the global patch $P_{i}$ is classified as a smooth patch and added to the set $S=\left\{s_{1},s_{2},\cdots s_{t}\right\}$.

So, the noise pool Ω can be derived by

(3) $$\Omega =\underset{i\in \left\{1,2,\cdots ,t\right\}}{\mathrm{collect}}\left(s_{i}-Mean\left(s_{i}\right)\right)$$

where $\mathrm{collect}\left(\cdot \right)$ denotes the noise collection operation. Notably, we perform the anisotropic Gaussian kernel on the paired synthetic data to simulate the generation of real noise. For unpaired data, the KernelGAN is regarded as a degraded kernel to estimate the distribution of the image noise under the constraints of Equation (4).

(4) $$G\left(I_{LR}\right)=\arg\min_{G}\max_{D}\left\{\left.\underset{x\in patches(I_{LR})}{E}\left[{\left(D(X)-1\right)}^{2}+D{\left(G\left(x\right)\right)}^{2}\right]+R\right\}\right.$$

where *R* is regular terms, *X* is image patch, and $I_{LR}$ is the input LR images.

Generally, the characteristics of the image manifest differently at different frequency spaces [24,48]. The content of the image is mainly expressed in its low-frequency components, while details, contours, noise, etc., are mainly expressed in high-frequency components. Based on this concept, we perform the convolutional Gaussian filter on the LR input to extract high- and low-frequency features. This stage can be formulated as

(5) $$\left\{\begin{array}{c} X_{L}=W_{L,\mathrm{Gaussian}}*X_{\mathrm{LR}}\\ X_{H}=X_{\mathrm{LR}}-X_{L} \end{array}\right.$$

where $X_{\mathrm{LR}}$ is the input LR image, and $X_{L}$ and $X_{H}$ are low- and high-frequency features of the input. $W_{L,\mathrm{Gaussian}}$ represents the convolutional Gaussian filter. ∗ represents the convolution operation. After that, we utilize the FCPG to process these high- and low-frequency components to make details clearer and remove inherent noise simultaneously. Finally, the generated images are further fed into the IPD for evaluating image quality.

### 3.3. Frequency Collaborative Progressive Generator

In our carefully designed PUGAN, the FCPG is used to generate HR images with clearer details and visually comfortable experience, and the structure of our FCPG is shown in Figure 1. The FCPG employs the RMB to explore multiscale features and the CAM to explore the complementarity and correlation of high/low-frequency features.

**Figure 2:**
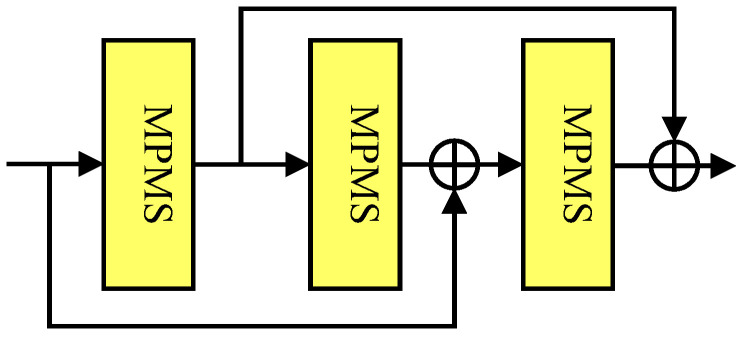
The structure of the RMB containing stacked three MPMSs using the dual-residual path.

**RMB:** The characteristics of the image in different scale spaces manifest differently. Hence, we design a high-efficiency RMB [see Figure 2] by stacking mixed-pooling multiscale-structure (MPMS) blocks in a dual-residual path manner (reported in [49]), which can make full use of the intermediate features.

Specifically, the features extracted by the $j_{th}$ MPMS block in the $i_{th}$ RMB can be expresed as

(6) $$F_{i,j}=H_{i,j}^{\mathrm{MPMS}}\left(F_{i,j-1}\right)$$

where $H_{i,j}^{\mathrm{MPMS}}\left(\cdot \right)$ denotes the $j_{th}$ MPMS blocks in the $i_{th}$ RMB. $F_{i,j}$ and $F_{i,j-1}$ are the output of $j_{th}$ and ${j-1}_{th}$ MPMS block in the $i_{th}$ RMB. After that, we further perform the dual-residual path to fully explore the intermediate informative features, namely

(7) $$F_{i}=F_{i,j-1}+F_{i,j+1}\left(F_{i-1}+F_{i,j}\right)$$

where $F_{i}$ and $F_{i-1}$ denote the output generated by the $i_{th}$ and ${i-1}_{th}$ RMBs. This carefully designed dual-residual path connection can guarantee more information to be bypassed.

**Figure 3:**
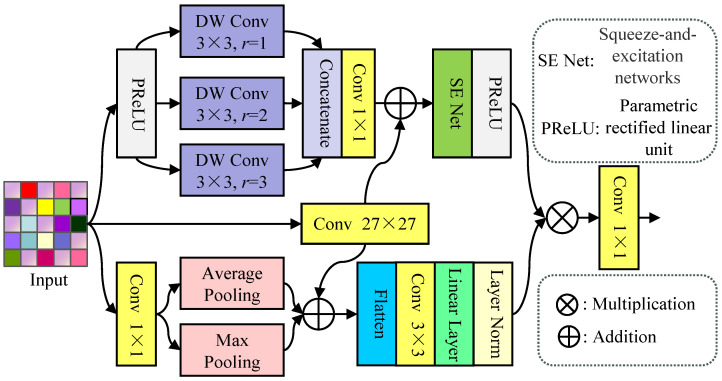
The structure of the MPMS, which is a trib-branch structure. The top branch mainly consists of depthwise convolution with different dilation rates, the middle branch only consists of a convolution layer with the size of 27×27, and the bottom branch mainly consists of average and max pooling. These carefully designed structures can fully explore multiscale features at local or global levels.

MPMS: The image features exhibit different representations at different scales and levels. Therefore, we employ the MPMS, a trip-branch structure (see Figure 3), to fully dig up such multiscale and hierarchical features. In the top branch, we first perform the parametric rectified linear unit (PReLU) function on the input, and further concatenate multiscale features extracted by a depthwise convolution with a dilation rate $r\in \left\{1,2,3\right\}$. This stage can be defined as

(8) $${F^{\prime }}_{\mathrm{in}}=\mathrm{cat}\left(\underset{r\in \left\{1,2,3\right\}}{{\mathrm{DWConv}}_{3}^{r}}\left(H_{\mathrm{PReLU}}\left(F_{\mathrm{in}}\right)\right)\right)$$

where $\underset{r\in \left\{1,2,3\right\}}{{\mathrm{DWConv}}_{3}^{r}}\left(\cdot \right)$ represents a depthwise convolution with a dilation rate $r\in \left\{1,2,3\right\}$, and $H_{\mathrm{PReLU}}\left(\cdot \right)$ denotes parametric rectified linear unit function. Subsequently, the output is processed by the 1×1 Conv, and further adds the feature extracted by the 27×27 Conv to it. Finally, the added result is processed by the squeeze-and-excitation networks (SE Net) block and PReLU to obtain the multiscale features ${F^{\prime }}_{\mathrm{multi}}$, i.e.,

(9) $${F^{\prime }}_{\mathrm{multi}}=H_{\mathrm{PReLU}}\left(H_{\mathrm{SE}}\left({\mathrm{Conv}}_{1}\left({F^{\prime }}_{\mathrm{in}}\right)⊕{\mathrm{Conv}}_{27}\left(F_{\mathrm{in}}\right)\right)\right)$$

where ${\mathrm{Conv}}_{1}\left(\cdot \right)$ and ${\mathrm{Conv}}_{27}\left(\cdot \right)$ denote a convolution withe the size of 1×1 and 27×27, respectively. $H_{\mathrm{SE}}\left(\cdot \right)$ denotes the SENet block.

In the bottom branch, we first perform 1×1 Conv to extract low-level features, and they are fed into average and max pooling to dig up hierarchical features. After that, these features are added to the features yielded by the 27×27 Conv, i.e.,

(10) $${F^{\prime }}_{\mathrm{hie}}=H_{\mathrm{AP}}\left({\mathrm{Conv}}_{1}\left(F_{\mathrm{in}}\right)\right)⊕H_{\mathrm{MP}}\left({\mathrm{Conv}}_{1}\left(F_{\mathrm{in}}\right)\right)⊕{\mathrm{Conv}}_{27}\left(F_{\mathrm{in}}\right)$$

where $H_{\mathrm{AP}}\left(\cdot \right)$ and $H_{\mathrm{MP}}\left(\cdot \right)$ denote average and max pooling, respectively. Finally, we employ successive Flatten, 3×3 Conv, Linearization, and Normalization to generate hierarchical features. And we further integrate hierarchical and multiscale features using the pixel multiplication to generate the mixed features $F_{\mathrm{mix}}$.

(11) $$F_{\mathrm{mix}}={F^{\prime }}_{\mathrm{mul}}⊗{F^{\prime }}_{\mathrm{hie}}$$

where ⊗ stands for the multiplication. Overall, the RMB employs the MPMS to multiscale hierarchical features, and the dual-patch skip connection is used to stack three MPMS to make full use of multilevel features.

**Figure 4:**
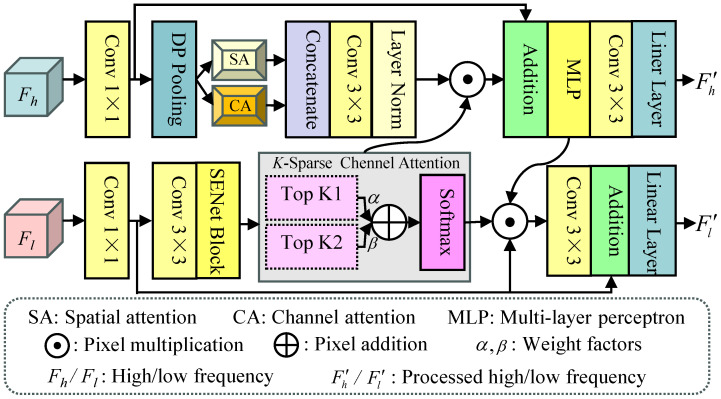
The structure of the CAM, and it consists of two branches for exploring the complementary nature of high/low-frequency information.

**CAM:** In our designed FCPG, the CAM is used to fully explore the complementarity and correlation of high-frequency and low-frequency characteristics. The structure of the CAM is illustrated in Figure 4, which is a dual-branch structure containing high- and low-frequency processing branch. For the high-frequency information, we first employ the 1×1 Conv and detail-preserving pooling to extract features with abundant details, and further fed them into the parallel channel attention (CA) and spatial attention (SA) to explore the correlation of features at the channel and spatial level. And we concatenate them for aggregating these features and further processed by the 3×3 Conv and normalization layer, i.e.,

(12) $$\left\{\begin{array}{c} {\hat{F}}_{h}=\mathrm{cat}\left(H_{\mathrm{SA}}\left({\mathrm{Conv}}_{1}\left(H_{\mathrm{DP}}\left(F_{h}\right)\right)\right),H_{\mathrm{CA}}\left({\mathrm{Conv}}_{1}\left(H_{\mathrm{DP}}\left(F_{h}\right)\right)\right)\right)\\ {\hat{F}}_{h}^{\prime }={\mathrm{Conv}}_{3}\left(H_{\mathrm{Norm}}\left(F_{h}\right)\right) \end{array}\right.$$

where $H_{\mathrm{DP}}\left(\cdot \right)$ denotes the detail preserving pooling. $H_{\mathrm{CA}}\left(\cdot \right)$ and $H_{\mathrm{SA}}\left(\cdot \right)$ stand for the channel attention (CA) and spatial attention (SA), respectively. $H_{\mathrm{Norm}}\left(\cdot \right)$ denotes the normlization layer. ${\mathrm{Conv}}_{3}\left(\cdot \right)$ denotes a convolution with the size of 3×3. After that, the weight $F_{\mathrm{Kspa}}^{l}$ yielded by the *K*-spaCA from the low-frequency branch multiplied by it, and the high frequency information $F_{h}$ processed by the 1×1 Conv is added. Finally, the output is processed by the successive MLP, 3×3 Conv, and linear layers to generate the final high-frequency features ${F^{\prime }}_{h}$, and it can be formulated as

(13) $${F^{\prime }}_{h}=H_{\mathrm{Linear}}\left({\mathrm{Conv}}_{3}\left(H_{\mathrm{MLP}}\left(\left({\hat{F}}^{\prime }⊙F_{\mathrm{Kspa}}^{l}\right)⊕{\mathrm{Conv}}_{1}\left(F_{h}\right)\right)\right)\right)$$

where ⊕ and ⊙ represent pixel addition and multiplication, $H_{\mathrm{MLP}}\left(\cdot \right)$ denotes the multi-layer perceptron, and $H_{\mathrm{Linear}}\left(\cdot \right)$ denotes linear layer.

For the low-frequency information, we employ the successive 1×1 Conv, 3×3 Conv, and SENet block to extract low-level features from the low-frequency information ${\hat{F}}_{l}$, and the output is further fed to the *K*-Sparse channel attention (*K*-spaCA). The *K*-spaCA employed two scoring strategies to select the most important $K_{1}$ and $K_{2}$ channels, and we further add them by weight factors α and β (empirically set them as 0.1 and 0.3, respectively.) and processed by the softmax. Subsequently, the output $F_{\mathrm{Kspa}}^{l}$, the feature ${\mathrm{Conv}}_{1}\left(F_{l}\right)$ extracted by the 1×1 Conv from the high frequency information $F_{h}$, and the features $F_{\mathrm{MLP}}^{h}$ extracted by the MLP are integrated by the pixel multiplication operation. This stage can be expressed as

(14) $$\left\{\begin{array}{c} {\hat{F}}_{l}=H_{\mathrm{SE}}\left({\mathrm{Conv}}_{3}\left({\mathrm{Conv}}_{1}\left(F_{l}\right)\right)\right)\\ F_{\mathrm{Kspa}}^{l}=H_{\mathrm{soft}}\left(0.1F_{K_{1}}\left({\hat{F}}_{l}\right)⊕0.3F_{K_{2}}\left({\hat{F}}_{l}\right)\right)\\ {\hat{F}}^{\prime }=F_{\mathrm{Kspa}}^{l}⊙{\mathrm{Conv}}_{1}\left(F_{l}\right)⊙F_{\mathrm{MLP}}^{h} \end{array}\right.$$

where $H_{\mathrm{soft}}\left(\cdot \right)$ denotes the softmax function. Finally, the integrated features ${\hat{F}}^{\prime }$ is processed by the 3×3 Conv, and further add it to the feature extracted by the 1×1 Conv from the high-frequency information $F_{h}$. The final low-frequency features can be obtained by the linear operation, i.e.,

(15) $${F^{\prime }}_{l}=H_{\mathrm{Linear}}\left({\hat{F}}^{\prime }⊕{\mathrm{Conv}}_{1}\left(F_{l}\right)\right)$$

where ${F^{\prime }}_{l}$ is the final low-frequency feature. In all, the CAM is a dual-branch structure, which can fully explore the complementary and correction of features at different frequency levels. The top branch mainly contains detail-preserving pooling (DP Pooling), self-attention (SA), and channel attention (CA) used to process the high-frequency information. Meanwhile, the bottom branch mainly contains SENet and K-spare channel attention to enhance the low-frequency information.

### 3.4. Image Perceptual Discriminator

**Figure 5:**
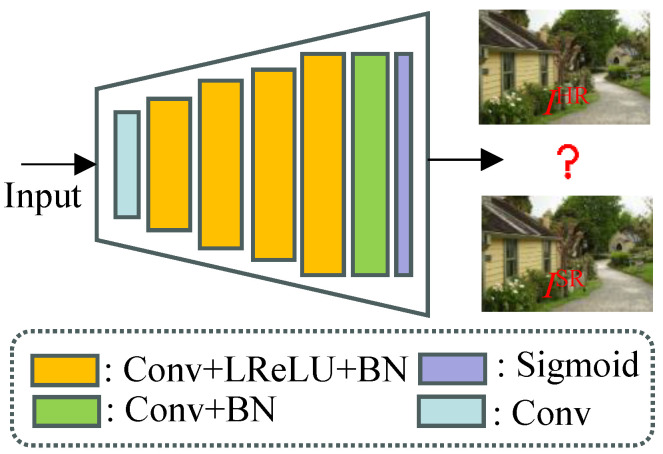
The archetecture of the disciminator used in our PUGAN.

In GAN-based methods, the discriminator plays a crucial role, which can assess the reconstructed image quality to drive the generated image quality toward reality. Hence, we design an image perceptual discriminator in accordance with [50] to solve the maximization problem in Equation (1). The structure of our discriminator is shown in Figure 5. It can easily be seen that our design is inspired by the architecture of the VGG network, and our discriminator mainly contains four convolutional layers with a increasing number of filter kernels. First, the convolutional layer is used to obtain shallow features. Then, we employ the strided convolutions to increase the image resolution each time. Finally, the feature maps of results are further processed by successive convolutional layer, batch normal (BN), and a sigmoid activation function to obtain a probability for sample classification.

### 3.5. Loss Function

For training our proposed PUGAN, we introduce a set of differentiable loss functions to guarantee the reconstructed HR images with clearer details and visually pleasing appearance.

Adversarial loss. The adversarial loss can encourage the network to produce solutions that reside on the natural image manifold by attempting to fool the discriminator. We formally define the adversarial loss across based on the probabilities of the FCPG for all training images,

(16) $$L_{\mathrm{adv}}=\sum_{n=1}^{N}-\log\left(D_{\theta_{D}}\left(G_{\theta_{G}}\left(I^{\mathrm{HR}}\right)\right)\right)$$

where *N* is the total number of training images, and $D_{\theta_{D}}\left(G_{\theta_{G}}\left(I^{\mathrm{LR}}\right)\right)$ denotes the probability that the reconstructed image is a visually pleasing HR image.

Spatial consistency loss. The pixel-wise MSE loss widly used in learning-based SISR methods, while the reconstructed HR images typically exihibit perceptually unsatisfying and overly smooth textures. Following Ledig et al. [50], we define the spatial consistency loss $L_{\mathrm{spa}}$ based on the ReLU activation layers of the pre-trained 19-layer VGG network $\phi_{\mathrm{VGG}}\left(\cdot \right)$ to measure the difference in neighboring regions between the reconstructed HR image *Y* and its corresponding LR version *I*. The $L_{\mathrm{spa}}$ can be defined as

(17) $$L_{\mathrm{spa}}=\frac{1}{K}\sum_{i=1}^{K}\sum_{j\in \Omega \left(i\right)}{\left(\left|\phi_{\mathrm{VGG}}\left(Y_{i}\right)-\phi_{\mathrm{VGG}}\left(Y_{j}\right)\right|-\left|\phi_{\mathrm{VGG}}\left(I_{i}\right)-\phi_{\mathrm{VGG}}\left(I_{j}\right)\right|\right)}^{2}$$

where *K* is the number of local areas. $\Omega \left(i\right)$ is the window size centered at the region *i*, and its size typically set as 4×4.

Color constancy loss. To make our reconstructed HR images exhibit better color fidelity, we introduce the color constancy loss function. This function relying on the Gray-World color constancy hypothesis can fully explore the relationships among R, G, and B channels of the reconstructed images. The $L_{\mathrm{col}}$ can be defined as

(18) $$L_{\mathrm{cor}}=\sum_{\forall \left(p,q\right)\in \varepsilon }{\left(J^{p}-J^{q}\right)}^{2},\varepsilon \in \left\{\left(R,G\right),\left(R,B\right),\left(G,B\right)\right\}$$

where $J^{p}$ and $J^{q}$ denote the average pixels value of channel *p* and *q* in reconstructed images.

Perceptual loss. It can assess a solution with respect to perceptually relevant characteristics. Hence, the perceptual loss formulated as a weighted sum of a spatial consistency loss, a color constancy loss, and an adversarial loss component can assess the quality of HR images from the aspects of spatial structure and color. The $L_{\mathrm{Per}}$ can be expressed as

(19) $$L_{\mathrm{Per}}=L_{\mathrm{spa}}+\lambda_{1}L_{\mathrm{col}}+\lambda_{2}L_{\mathrm{adv}}$$

where the weights $\lambda_{1}$ and $\lambda_{2}$ are applied to balance the details enhancement and color correction, empirically setting them as 0.1 and $10^{-3}$, respectively.

## 4. Experimental Results and Analysis

This section first presents the implementation details and experimental setting. Subsequently, the validation experiments, ablation study, generalization, and computational complexity are demonstrated. Finally, we discuss the limitations and future works related to our PUGAN.

### 4.1. Implementation Details

Four common publicly used benchmarks, including three paired datasets—NTIRE 2020 [51], Urban 100 [52], and B 100 [53]—and a real-world dataset DPED [54], are used in our work. Among them, three paired public datasets contain synthesized RGB images, and we perform the anisotropic Gaussian kernel on them to simulate the generation of real noise. The DPED [54] dataset contains unpaired images captured by a smartphone, and we apply the KernelGAN on DPED [54] to estimate the distribution of the noise. During the training, we randomly select 80% images from NTIRE 2020 [51], and the remaining 20% of the data is the test setting. Additionally, we further perform our PUGAN and other state-of-the-art SISR methods on the Urban 100 [52], B 100 [53], and DPED [54] datasets to verify their effectiveness in real-world applications.

In our validation experiments, we employ the commonly used technologies, including rotating by 90°, horizontal flipping, and scaling, to augment the training datasets (randomly select 80% images from NTIRE 2020 [51]) containing RGB images. The input RGB images are randomly cropped into a specific size of 64×64. Our proposed PUGAN and other compared SISR methods are implemented in the Pytorch framework on an NVIDIA Tesla P100 GPU to test their performance. For better training of our SISR method, the batch size is set to 16, the number of iterations is set to 60,000, and the learning rate is set to 1 × 10^−4^. We further use the ADAM optimizer with $\beta_{1}=0.5$, $\beta_{2}=0.999$, and $\varepsilon =10^{-8}$ to optimize the parameters of our carefully designed PUGAN.

### 4.2. Experimental Settings

Comparison Methods: We select some state-of-the-art SISR approaches as comparison methods to verify the performance of our proposed PUGAN for the SISR vision task. These compared methods contain ESRGAN [27], SDSR [48], TDSR [48], RealSR [55], DASR [56], DUSGAN [28], IDMBSR [57], FASR [58], DMGSR [59], and SRMamba-T [26]. To ensure the fairness of the comparison, all above-listed SISR comparison methods adopt publicly available source code with recommended parameters.

Evaluation Metrics: Three commonly used evaluation metrics including peak signal-to-noise ratio (PSNR), structural similarity index (SSIM), and learned perceptual image patch similarity (LPIPS) are applied to the quantitative assessment of image quality. And these metrics can be calculated by

(20) $$\mathrm{PSNR}=10\times \log_{10}\frac{max^{2}\left(\hat{I}\right)}{\mathrm{MSE}\left(I,\hat{I}\right)}$$

where I and $\hat{I}$ are the ground truth and reconstructed HR images. $\mathrm{MSE}\left(\cdot \right)$ represents the solution of variance, and $max^{2}\left(\hat{I}\right)$ denotes the maximum pixel of reconstructed HR images $\hat{I}$.

(21) $$\mathrm{SSIM}=\frac{\left(2\mu_{x}\mu_{y}+C_{1}\right)\left(2\sigma_{xy}+C_{2}\right)}{\left(\mu_{x}^{2}+\mu_{y}^{2}+C_{1}\right)\left(\sigma_{x}^{2}+\sigma_{y}^{2}+C_{2}\right)}$$

where $\mu_{x}$ and $\mu_{y}$ denote the average pixels value of the ground truth I and reconstructed HR image $\hat{I}$. $\sigma_{x}$ and $\sigma_{y}$ are the pixel variances of these two images, and $\sigma_{xy}$ is the pixel covariance. $C_{1}$ and $C_{2}$ are constant.

(22) $$\mathrm{LPIPS}=\sum_{l}\frac{\sum_{h,w}{∥w_{l}⊙\left(y_{hw}^{l}-{\hat{y}}_{hw}^{l}\right)∥}_{2}^{2}}{H^{l}W^{l}}$$

where $y_{hw}^{l}$ and ${\hat{y}}_{hw}^{l}$ denote the input and reconstructed images’ features of the $l_{th}$ layer in the trained CNN-based model. H×W is the height and width of the image.

Among them, PSNR can measure the differences at the pixel-level, and a higher PSNR score suggests a better quality while exhibiting relatively weak correlation with human visual perception. SSIM can measure the similarity between reconstructed HR results and their corresponding ground truth in terms of brightness, contrast, and structure, and a higher SSIM score suggests a better structure preservation. LPIPS is a learning-based image quality assessment method, which can measure the differences in feature maps extracted by a pre-trained CNN model. A lower LPIPS score suggests a better visual perception.

### 4.3. Comparisons on the Synthetic Datasets

We first perform our PUGAN and compared SISR approaches on the Urban 100 [52], B 100 [53], and DPED [54] datasets to test their performance in the SISR task from the qualitative and quantitative evaluations.

**Figure 6:**
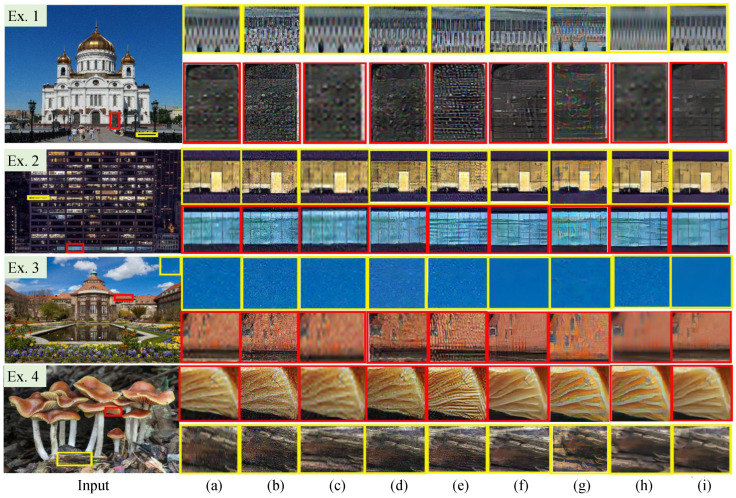
Vision comparison of different SISR methods on the NTIRE 2020 [51]. The HR image reconstructed by (**a**) ESRGAN [27], (**b**) SDSR [48], (**c**) TDSR [48], (**d**) RealSR [55], (**e**) DASR [56], (**f**) IDMBSR [57], (**g**) FASR [58], (**h**) DMGSR [59], and (**i**) Ours.

Qualitative Evaluation. Figure 6 demonstrates the HR images randomly selected from the NTIRE 2020 [51]. We can easily observe that SDSR [48] and FASR [58] inevitably introduce observable noise in some reconstructed HR images (e.g., the first row of the Ex. 1 and Ex. 4 in Figure 6). In addition, DASR [56], FASR [58], and IDMBSR [57] can make the details clearer for some reconstructed HR images, but they fail in removing artifact-halos. FASR [58] also shows poor performance in noise suppression. DASR [56] yields unnatural visual experience (the first row of the Ex. 4 in Figure 6). ESRGAN [27] and TDSR [48] can generate noise-free HR images. However, these two SISR methods are often considered inferior to other compared SISR models in detail enhancement. DMGSR [59] shows better performance in removing significant noise, while it is unsatisfactory for local details and edges preservation (e.g., the first row of the Ex. 1 in Figure 6, and the second row of the Ex. 3 in Figure 6). In contrast, our PUGAN can effectively yield visually satisfactory and noise-free HR images with clearer details and edges.

Quantitative Evaluation. Table 1 presents the average PSNR, SSIM, and LPIPS scores of different SISR approaches for ×2 and ×4 on the Urban 100 [52], B 100 [53], and DPED [54] datasets. From the quantitative evaluation scores of three public benchmarks in Table 1, we can easily find that our PUGAN yields comparable and higher values of PSNR and SSIM than compared SISR methods. For the LPIPS, the proposed PUGAN outperforms other listed state-of-the-art SISR models, suggesting that our method can reconstruct HR images with a pleasant visual experience. Overall, the qualitative and quantitative comparisons demonstrate that our carefully designed PUGAN exhibits superior performance for generating noise-free and artifact-free images with clearer details.

### 4.4. Comparisons on the Real-World Datasets

To verify the application on real-world data, we further perform all the above-mentioned SISR approaches on the DPED [54] dataset and assess their performance from both quantitative and qualitative perspectives.

**Figure 7:**
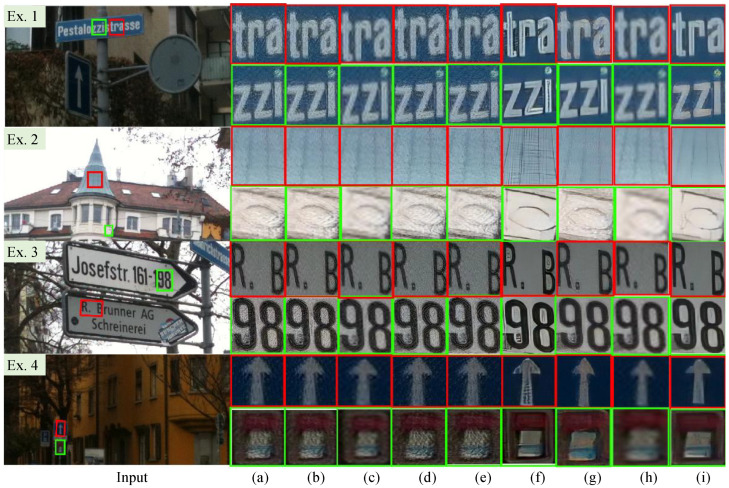
Vision comparison of different SISR methods on the DPED [54]. The HR image reconstructed by (**a**) ESRGAN [27], (**b**) SDSR [48], (**c**) TDSR [48], (**d**) RealSR [55], (**e**) DASR [56], (**f**) IDMBSR [57], (**g**) FASR [58], (**h**) DMGSR [59], and (**i**) Ours.

Qualitative Evaluation. We show the HR images reconstructed by our PUGAN and compared SISR methods in Figure 7. As easily observed from the reconstructed HR results, ESRGAN [27], SDSR [48], RealSR [55], and DASR [56] introduce observable noise into some HR images. In addition, RealSR [55] fails in making details and edges clearer. TDSR [48] is unsuccessful in removing hazy-like appearance and noise suppression (e.g., the first row of the Ex. 1 in Figure 7, and the second row of the Ex. 2 in Figure 7). IDMBSR [57] shows satisfactory performance in detail enhancements but yields undesired artifact-halos in some HR images (e.g., the second row of the Ex. 3 in Figure 7). DMGSR [59] cannot make the details and contours clearer, with some DMGSR-reconstructed HR images suffering from blurry details (e.g., the first and second row of the Ex. 4 in Figure 7) and hazy-like appearance (e.g., the second row of the Ex. 2 in Figure 7). In comparison, our carefully designed PUGAN is superior to the compared SISR methods in detail enhancement and noise suppression, and it also works better in removing artifact-halos and hazy-like appearance.

**Quantitative Evaluation.** We also perform the LPIPS to evaluate the HR images reconstructed by different SISR methods, and their average LPIPS score for the DPED [54] dataset are illustrated in Table 2. From Table 2, it can easily be observed that our PUGAN can yield more satisfactory and comparable LPIPS scores than compared SISR approaches. The quantitative and qualitative assessment results suggest that our proposed method achieves the state-of-the-art performance for the SISR vision task.

### 4.5. Ablation Study

To fully test the effect of each component in our PUGAN, we perform ablation studies including frequency decomposition (FD), upsampling operations (UO), and CAM on the test datasets.

**Figure 8:**
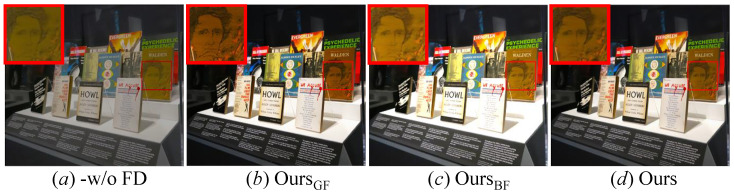
Vision comparison of our PUGAN with different FD operations. (**a**) -*w/o* FD, (**b**) ${\mathrm{Ours}}_{\mathrm{GF}}$, (**c**) ${\mathrm{Ours}}_{\mathrm{BF}}$, (**d**) Ours.

**Figure 9:**
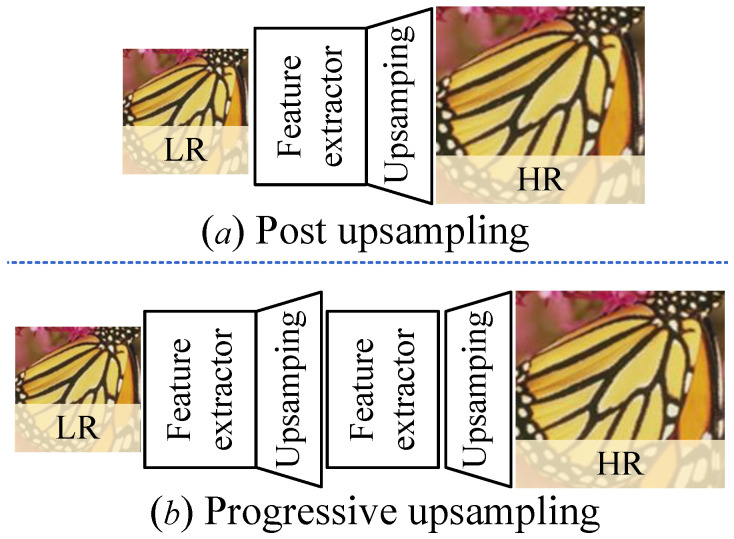
The flowchart of post upsampling (**a**) and progressive upsampling (**b**).

**Figure 10:**
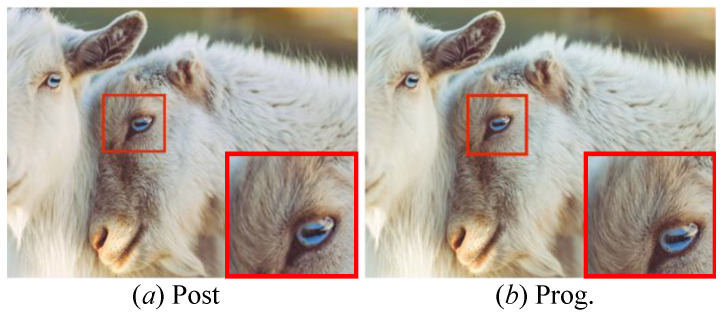
Vision comparison of our PUGAN with different UO. Among them, the results yielded by (**a**) Post and (**b**) Prog. upsampling operations.

**Figure 11:**
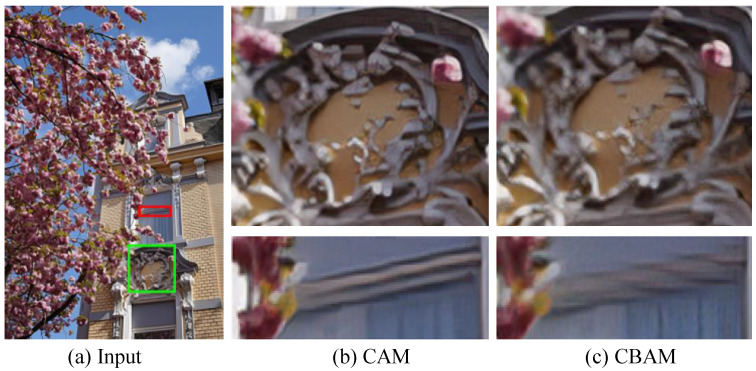
(**a**) Input, Vision comparison of our proposed PUGAN with (**b**) CAM and (**c**) CBAM.

(1) Study of the FD: The FD operation can separate noise, details, and content from the LR images. For verifying its effectiveness, we first remove the FD operation from our PUGAN (-*w/o* FD), then the guided filter (GF) and bilateral filter (BF) are used to extracted frequency features (${\mathrm{Ours}}_{\mathrm{GF}}$ and ${\mathrm{Ours}}_{\mathrm{BF}}$). Figure 8 presents HR images generated by our method with different FD operations. It can be observed that -*w/o* FD fails in details boosting. In contrast, our PUGAN can generate more visually pleasing images with clearer details than other FD operations. Furthermore, we present average PSNR and SSIM scores of our PUGAN with different FD operations in Table 3. It can easily be found that our method yields higher PSNR and SSIM scores than ablated models. Overall, the qualitative and quantitative analyses show that the FD operation plays an indispensable role in our designed PUGAN.

(2) Study of the UO: Figure 9 presents the flowcharts of post-upsampling and progressive upsampling, named post and prog., respectively. To analyze their effectiveness, we perform our PUGAN with different UO on test datasets, and the generated HR images are demonstrated in Figure 10. It can easily be seen that our PUGAN with prog. means can make local details clearer than the post means. The likely reason is that the prog. means with partial skip connections can alleviate information loss and ambiguity. We further present their PSNR and SSIM scores in Table 3. Intuitively, our method with prog. means outperforms post means in yielding higher PSNR and SSIM scores.

(3) Study of the CAM: Figure 11 presents the HR images yielded by our PUGAN with CAM and CBAM. It can be observed that our PUGAN with CAM can generate visually comfortable images with clearer details. Furthermore, their PSNR and SSIM scores are presented in the Table 4. We can also find that the CAM can bring absolute improvements in PSNR and SSIM scores for our SISR method. That is to say, our PUGAN with CAM outperforms the latter in terms of qualitative and quantitative assessments, benefiting from our designed CAM.

**Figure 12:**
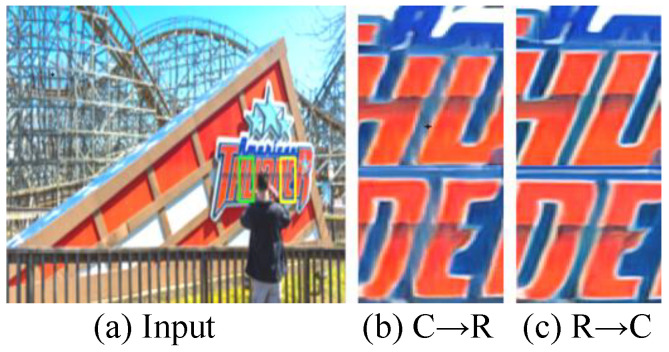
Vision comparison of our proposed PUGAN with defferent combinations. (**a**) Input, the reconstructed HR images by (**b**) C → R (CAB-RMB) and (**c**) R → C (RMB-CAB).

There are two main combinations of the sequence of CAM and RMB, including C → R (CAB-RMB) and R → C (RMB-CAB). For determining the optimal combination sequence, we evaluate them on the test dataset and their HR images are shown in Figure 12. From Figure 12, we can easily see that the latter combination, i.e., R → C (RMB-CAB), can make HR images exhibit clearer details and outlines. Table 4 further illustrates their corresponding PSNR and SSIM scores. We observe that R → C (RMB-CAB) outperforms C → R (CAB-RMB) in PSNR and SSIM evaluation metrics. In conclusion, the qualitative and quantitative analyses have demonstrated that the R → C (RMB-CAB) is the optimal combination sequence.

### 4.6. Comparison of Computational Complexity

The computational complexity of learning-based applications determines their real-world applications. Hence, we compare our carefully designed PUGAN with other state-of-the-art SISR methods in terms of Parameters and Flops to verify their work efficiency. Their parameters (Param) and Flops are demonstrated in Table 5. From Table 5, it can be easily observed that most compared SISR methods exhibit more Parameters and Flops, leading to a heavy computational burden and a narrow practical application. By contrast, our proposed PUGAN enjoys fewer parameters and higher inference speed than the above-listed SISR methods. This result mainly occurs because the progressive upsampling can decompose large-scale input into smaller-scale ones to reduce the model’s learning complexity.

### 4.7. Application on Pathological Images

Digital pathological images are the gold standard for cancer diagnosis, which contain tumor microenvironment (TME), including tumor epithelial, tumor-infiltrating lymphocytes (TILs), tumor-associated stroma, etc., clinically related to the occurrence, development, and metastasis of tumors [8,60,61]. However, the scanning of high-magnification pathological image is time-consuming and difficult to store, limiting its application in clinical diagnosis. To address this issue, we train our carefully designed PUGAN on the HistoSR dataset [8] to examine its generalization. We divided the HistoSR dataset at a ratio of 7:3 into the training and test sets, and we set the initial parameters of our PUGAN to the previously determined values. Notably, all mentioned comparison methods are retrained in the same way as our proposed model.

**Figure 13:**
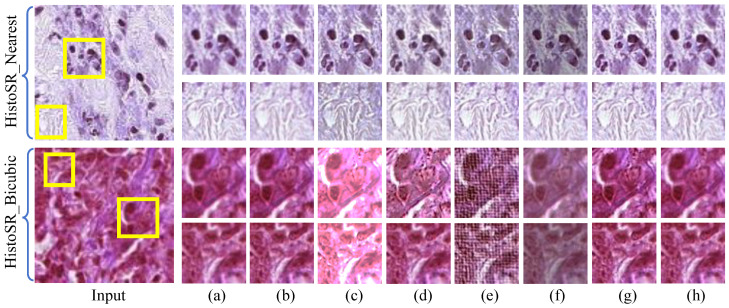
Vision comparison of our PUGAN on the HistoSR [8] with the nearest and bicubic degradations. The HR pathological images reconstructed by (**a**) SDSR [48], (**b**) RealSR [55], (**c**) DASR [56], (**d**) IDMBSR [57], (**e**) FASR [58], (**f**) DMGSR [59], (**g**) Ours, and (**h**) Ground Truth.

Figure 13 presents the HR pathological image patches generated by our PUGAN and other state-of-the-art SISR methods. The following observation can be seen from Figure 13: Most SISR methods struggle to yield visually satisfactory HR pathological image patches, and some generated HR images confront artifact-halos and blurry details [e.g., the bottom picture of Figure 13c]. Additionally, FASR [58] and DMGSR [59] are unsuitable for removing inherent noise. DASR [56] reconstructs HR pathological image patches, while introducing an uncomfortable visual experience. In contrast, the proposed PUGAN effectively removes artifact-halos and makes structural details clearer with the benefit of our carefully designed method. Additionally, we also present the average PSNR and SSIM scores of different SISR methods in Table 6. It can be seen that our PUGAN is superior to other compared SISR methods in generating more satisfactory PSNR and SSIM scores. Overall, the quantitative and qualitative evaluations suggest that our carefully designed SISR approach consistently produces noise-free and artifact-free HR pathological image patches with observable and clearer details. The experiments further demonstrate that our method exhibits robust generalization ability.

### 4.8. Limitation and Discussion

**Figure 14:**
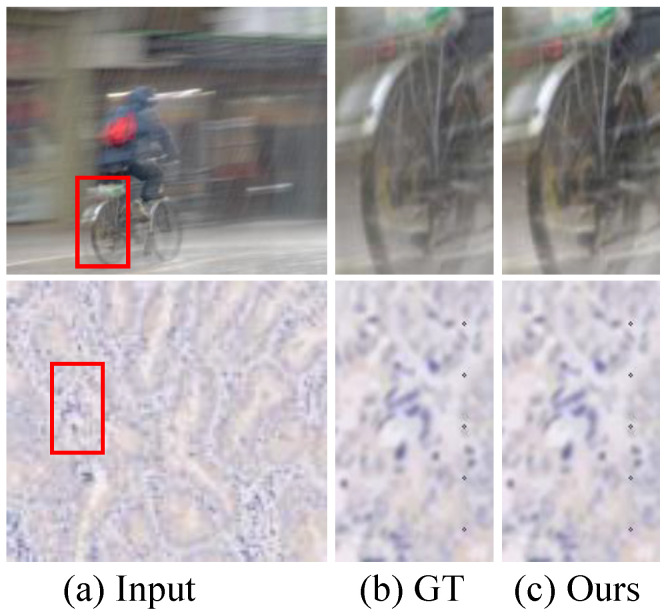
Some failure instances of our proposed PUGAN. From left to right, (**a**) Input, (**b**) Ground Truth (GT), and (**c**) Ours.

SISR plays a crucial role in low-level computer vision, which can yield a visually HR image from its corresponding LR version and further promote the performance of image content understanding, image classification, and other advanced computer vision tasks. Experiments have proven that our PUGAN works well on the SISR tasks for natural and pathological images in most situations. However, the proposed method fails in LR images with complex degradations, such as motion blur, severe compression artifacts, etc. For example, we present some failure instances reconstructed by our proposed PUGAN in Figure 14. It can easily be seen that the reconstructed HR images exhibit unsatisfactory details and undesired artifact-halos. The reason may be that our PUGAN only collects inherent noise from the LR input and does not consider the LR images captured in the real world, which exhibit multiple and complex degradation. In the future, we will inject promote learning and specialized mixture-of-experts (MoE) for tackling these challenging issues.

## 5. Conclusions

This paper proposes a feasible and effective SISR method based on the progressive upsampling generative adversarial network with collaborative attention named PUGAN. This method first employs a sliding window to collect noise from the inputs to construct a noise pool and further randomly selects noise to simulate real noise. Subsequently, the convolutional Gaussian filtering is used to extract the high- and low-frequency information of the LR input, and the FCPG further performs the residual multiscale blocks (RMBs) and collaborative attention mechanism (CAM) to fully explore the features at different scales and frequencies for yielding artifact-free and noise-free images with clearer details. Meanwhile, the progressive upsampling strategy is introduced to reduce the model’s complexity. Finally, the discriminator is used to judge the differences between the reconstructed images and their corresponding images for generating visually pleasing and artifact-free images with clearer details. Although our method shows better performance in the SISR task, it still cannot make the details clearer in the partial darkness of the image. We leave this challenging case as our future work.

## Figures and Tables

**Table 1:** Quantitative analysis of different comparison methods on paired benchmarks. Red/blue text stands for the best/second-best performance.

Methods	Scale	NTIRE 2020			Urban 100			B 100		
		PSNR	SSIM	LPIPS	PSNR	SSIM	LPIPS	PSNR	SSIM	LPIPS
ESRGAN [27]	×2	30.989_(±2.387)_	0.9179_(±0.1963)_	0.4435_(±0.1100)_	29.507_(±2.301)_	0.8946_(±0.2073)_	0.5107_(±0.1206)_	29.987_(±2.375)_	0.9056_(±0.1824)_	0.4789_(±0.1326)_
SDSR [48]	×2	31.218_(±2.112)_	0.9011_(±0.1788)_	0.3574_(±0.0999)_	31.075_(±2.300)_	0.8956_(±0.1998)_	0.3689_(±0.1203)_	31.173_(±2.212)_	0.8997_(±0.1314)_	0.3613_(±0.0954)_
TDSR [48]	×2	31.369_(±1.428)_	0.9237_(±0.1368)_	0.2917_(±0.1016)_	31.101_(±1.306)_	0.9063_(±0.1208)_	0.3104_(±0.1251)_	31.238_(±1.511)_	0.9113_(±0.1474)_	0.2988_(±0.1026)_
RealSR [55]	×2	32.015_(±1.289)_	0.9318_(±0.1710)_	0.2540_(±0.0310)_	31.997_(±1.355)_	0.9219_(±0.1604)_	0.2678_(±0.0294)_	32.000_(±1.747)_	0.9267_(±0.1567)_	0.2588_(±0.0234)_
DASR [56]	×2	30.586_(±1.575)_	0.9112_(±0.1603)_	0.2309_(±0.0177)_	30.054_(±1.462)_	0.9084_(±0.1412)_	0.2113_(±0.0237)_	30.510_(±1.428)_	0.9109_(±0.1379)_	0.2029_(±0.0199)_
DUSGAN [28]	×2	30.111_(±1.554)_	0.9099_(±0.1097)_	0.2619_(±0.0192)_	30.093_(±1.274)_	0.9001_(±0.1724)_	0.2310_(±0.0210)_	30.108_(±1.426)_	0.9085_(±0.1257)_	0.2403_(±0.0199)_
IDMBSR [57]	×2	31.268_(±1.274)_	0.9326_(±0.1667)_	0.2213_(±0.0174)_	31.001_(±1.363)_	0.9254_(±0.1248)_	0.2271_(±0.0169)_	31.217_(±1.578)_	0.9300_(±0.1780)_	0.2100_(±0.0210)_
FASR [58]	×2	32.385_(±1.302)_	0.9384_(±0.1107)_	0.2190_(±0.0134)_	32.107_(±1.425)_	0.9289_(±0.1007)_	0.2037_(±0.0348)_	31.589_(±1.478)_	0.9412_(±0.1694)_	0.2095_(±0.0484)_
DMGSR [59]	×2	32.867_(±1.177)_	0.9334_(±0.0989)_	0.1901_(±0.0235)_	31.271_(±1.546)_	0.9129_(±0.1001)_	0.1945_(±0.0197)_	31.968_(±1.701)_	0.9299_(±0.1092)_	0.1934_(±0.0218)_
SRMamba-T [26]	×2	33.247_(±1.439)_	0.9591_(±0.1027)_	0.1393_(±0.0472)_	33.103_(±1.538)_	0.9483_(±0.1230)_	0.1536_(±0.0589)_	33.119_(±1.672)_	0.9522_(±0.1196)_	0.1496_(±0.0503)_
Ours	×2	33.987_(±1.101)_	0.9673_(±0.0799)_	0.1210_(±0.0200)_	32.966_(±1.239)_	0.9483_(±0.0983)_	0.1431_(±0.0376)_	33.627_(±1.377)_	0.9546_(±0.0865)_	0.1354_(±0.0274)_
ESRGAN [27]	×4	23.745_(±2.179)_	0.6852_(±0.1207)_	0.2071_(±0.0989)_	23.379_(±2.237)_	0.6671_(±0.1331)_	0.2173_(±0.0899)_	23.401_(±2.101)_	0.6798_(±0.1123)_	0.2239_(±0.0783)_
SDSR [48]	×4	22.909_(±2.4810)_	0.6854_(±0.1179)_	0.4384_(±0.0899)_	22.764_(±2.324)_	0.6672_(±0.1228)_	0.4697_(±0.0675)_	22.849_(±2.3783)_	0.6917_(±0.1023)_	0.4462_(±0.0666)_
TDSR [48]	×4	21.998_(±2.079)_	0.4358_(±0.0998)_	0.4609_(±0.0418)_	21.590_(±2.111)_	0.4083_(±0.1001)_	0.4731_(±0.0098)_	21.783_(±1.985)_	0.4576_(±0.9597)_	0.4547_(±0.0107)_
RealSR [55]	×4	24.989_(±1.997)_	0.6919_(±0.0579)_	0.2270_(±0.0071)_	24.869_(±1.939)_	0.6704_(±0.0662)_	0.2346_(±0.0082)_	24.953_(±1.879)_	0.6888_(±0.0710)_	0.2241_(±0.0074)_
DASR [56]	×4	25.461_(±1.679)_	0.7992_(±0.1023)_	0.2013_(±0.0064)_	25.307_(±1.705)_	0.7597_(±0.1084)_	0.2039_(±0.0059)_	25.436_(±1.652)_	0.7793_(±0.1123)_	0.2010_(±0.0045)_
DUSGAN [28]	×4	24.218_(±1.811)_	0.6172_(±0.0992)_	0.5625_(±0.1998)_	24.192_(±1.707)_	0.5423_(±0.1010)_	0.5512_(±0.2000)_	24.203_(±1.910)_	0.5593_(±0.1869)_	0.5104_(±0.1118)_
IDMBSR [57]	×4	25.429_(±1.232)_	0.8079_(±0.1027)_	0.2000_(±0.0100)_	25.379_(±1.407)_	0.7540_(±0.1005)_	0.2017_(±0.0998)_	25.400_(±1.376)_	0.7998_(±0.0571)_	0.2028_(±0.0646)_
FASR [58]	×4	25.971_(±1.223)_	0.8113_(±0.1116)_	0.1996_(±0.0109)_	25.648_(±1.392)_	0.8023_(±0.1401)_	0.2208_(±0.0210)_	25.946_(±1.245)_	0.8099_(±0.1327)_	0.2175_(±0.0119)_
DMGSR [59]	×4	26.078_(±1.444)_	0.7782_(±0.1228)_	0.1979_(±0.0174)_	25.976_(±1.652)_	0.8347_(±0.1000)_	0.2002_(±0.0971)_	26.013_(±1.535)_	0.8498_(±0.1219)_	0.2113_(±0.0163)_
SRMamba-T [26]	×4	26.201_(±1.457)_	0.8603_(±0.1238)_	0.1980_(±0.0358)_	26.119_(±1.676)_	0.8417_(±0.2018)_	0.1999_(±0.0758)_	26.198_(±1.469)_	0.8541_(±0.1556)_	0.2054_(±0.0754)_
Ours	×4	26.349_(±1.139)_	0.8721_(±0.1015)_	0.1975_(±0.0236)_	26.110_(±1.458)_	0.8614_(±0.1298)_	0.1983_(±0.0307)_	26.306_(±1.367)_	0.8803_(±0.1201)_	0.1978_(±0.0286)_

**Table 2:** The average LPIPS scores of different SISR methods on the DPED [54] for the scale of ×2 and ×4. **Bold** text denotes the best performance.

**Scale**	**ESRGAN**	**SDSR**	**TDSR**	**RealSR**	**DUSGAN**
×2	0.3579_(±0.0311)_	0.2917_(±0.0296)_	0.3001_(±0.0248)_	0.2785_(±0.0415)_	0.2711_(±0.0340)_
×4	0.3784_(±0.0203)_	0.3037_(±0.0279)_	0.3209_(±0.0240)_	0.2699_(±0.0246)_	0.2868_(±0.0124)_
**Scale**	**IDMSBSR**	**FASR**	**DMGSR**	**SRMammba-T**	**Ours**
×2	0.2432 _(±0.0211)_	0.2347_(±0.0209)_	0.2137_(±0.0295)_	0.1758_(±0.0204)_	**0.1560**_(±0.0109)_
×4	0.2559_(±0.0191)_	0.2466_(±0.0158)_	0.2394_(±0.0100)_	0.2003_(±0.0097)_	**0.1884**_(±0.0089)_

**Table 3:** The average PSNR and SSIM scores of our PUGAN with different operations on the test datasets. **Bold** text stands for the best performance.

Operations	Scale	PSNR	SSIM	LPIPS
-*w/o* FD	×4	25.997_(±2.018)_	0.8104_(±0.1857)_	0.2317_(±0.0876)_
${\mathrm{Ours}}_{\mathrm{BF}}$	×4	26.010_(±1.488)_	0.8494_(±0.1540)_	0.2011_(±0.0548)_
${\mathrm{Ours}}_{\mathrm{GF}}$	×4	26.101_(±1.387)_	0.8500_(±0.1421)_	0.1998_(±0.0410)_
Ours	×4	**26.349**_(±1.139)_	**0.8721**_(±0.1015)_	**0.1975**_(±0.0236)_
Post	×4	26.001_(±1.569)_	0.8499_(±0.1526)_	0.2010_(±0.0483)_
Prog.	×4	**26.349**_(±1.139)_	**0.8721**_(±0.1015)_	**0.1975**_(±0.0236)_

**Table 4:** The average PSNR and SSIM scores of our PUGAN with different operations on the test datasets. **Bold** text stands for the best performance.

Methods	Scale	PSNR	SSIM	LPIPS
CAM	×4	**26.349**_(±1.139)_	**0.8721**_(±0.1015)_	**0.1975**_(±0.0236)_
CBAM	×4	26.001_(±1.786)_	0.8341_(±0.1128)_	0.2103_(±0.0672)_
C → R	×4	26.130_(±1.987)_	0.8512_(±0.1389)_	0.1987_(±0.0670)_
R → C	×4	**26.349**_(±1.139)_	**0.8721**_(±0.1015)_	**0.1975**_(±0.0236)_

**Table 5:** Computational complexity comparison of existing SISR methods. Red/blue text stands for the best/second-best performance.

Method	Param (M)	Flops (G)
ESRGAN [27]	16.71	12.02
SDSR [48]	0.02	0.15
TDSR [48]	6.20	2.01
RealSR [55]	1.70	5.30
DASR [56]	1.48	5.00
DUSGAN [28]	1.97	3.03
IDMBSR [57]	1.71	3.33
FASR [58]	1.28	6.80
DMGSR [59]	1.51	3.09
SRMamba-T [26]	1.26	4.22
Ours	0.23	1.72

**Table 6:** Quantitive comparison of our proposed PUGAN on the HistoSR with the nearest and bicubic degeradations. **Bold** text stands for the best performance.

Methods	Histo_Nearest			Histo_Bicubic		
	PSNR	SSIM	LPIPS	PSNR	SSIM	LPIPS
ESRGAN [27]	31.984_(±1.113)_	0.8867_(±0.0174)_	0.2496_(±0.0011)_	31.998_(±0.961)_	0.8718_(±0.1312)_	0.2319_(±0.0101)_
SDSR [48]	32.004_(±1.166)_	0.9287_(±0.0191)_	0.2667_(±0.0172)_	32.901_(±2.183)_	0.9413_(±0.0137)_	0.2579_(±0.0075)_
TDSR [48]	32.500_(±2.143)_	0.9238_(±0.1001)_	0.2419_(±0.0111)_	33.010_(±1.967)_	0.9589_(±0.1347)_	0.2274_(±0.0401)_
RealSR [55]	32.499_(±1.428)_	0.9301_(±0.1547)_	0.2398_(±0.0018)_	33.0299_(±1.557)_	0.9574_(±0.1168)_	0.2299_(±0.0121)_
DASR [56]	32.489_(±2.010)_	0.9000_(±0.0153)_	0.2501_(±0.0026)_	31.887_(±1.993)_	0.9153_(±0.0067)_	0.2243_(±0.0009)_
DUSGAN [28]	32.413_(±1.329)_	0.9236_(±0.0276)_	0.2517_(±0.0243)_	33.002_(±1.568)_	0.9564_(±0.0386)_	0.2287_(±0.0015)_
IDMBSR [57]	32.350_(±3.002)_	0.9148_(±0.1760)_	0.2418_(±0.0124)_	33.098_(±2.510)_	0.9583_(±0.1548)_	0.2235_(±0.0101)_
FASR [58]	32.217_(±1.111)_	0.8997_(±0.1007)_	0.2467_(±0.0201)_	32.763_(±0.989)_	0.9401_(±0.0984)_	0.2268_(±0.0115)_
DMGSR [59]	32.203_(±1.856)_	0.9023_(±0.1452)_	0.2971_(±0.0026)_	33.111_(±1.138)_	0.8999_(±0.1142)_	0.3105_(±0.0957)_
SRMamba-T [26]	32.323_(±1.859)_	0.9317_(±0.1046)_	**0.2370**_(±0.0115)_	33.104_(±1.642)_	0.9599_(±0.0984)_	0.2310_(±0.0710)_
Ours	**32.601**_(±1.010)_	**0.9398**_(±0.0842)_	0.2376_(±0.0257)_	**33.138**_(±0.979)_	**0.9609**_(±0.0271)_	**0.2217**_(±0.0004)_

## Data Availability

The data presented in this study are available in NTIRE dataset at https://aistudio.baidu.com/datasetdetail/216023 (accessed on 15 November 2025), reference number [44], Urban 100 dataset at https://tianchi.aliyun.com/dataset/88706/ (accessed on 15 November 2025), reference number [45], DPED dataset at https://aiff22.github.io/#dataset (accessed on 15 November 2025), reference number [46], and HistoSR dataset at https://pan.baidu.com/s/1H4TFsKNKZTno8Sz6IOs38A?pwd=7rqv (accessed on 15 November 2025), reference number [52].
